# Clotrimazole Preferentially Inhibits Human Breast Cancer Cell Proliferation, Viability and Glycolysis

**DOI:** 10.1371/journal.pone.0030462

**Published:** 2012-02-08

**Authors:** Cristiane M. Furtado, Mariah C. Marcondes, Mauro Sola-Penna, Maisa L. S. de Souza, Patricia Zancan

**Affiliations:** 1 Laboratório de Oncobiologia Molecular (LabOMol), Departamento de Fármacos, Faculdade de Farmácia, Universidade Federal do Rio de Janeiro, Rio de Janeiro, Rio de Janeiro, Brazil; 2 Laboratório de Enzimologia e Controle do Metabolismo (LabECoM), Departamento de Fármacos, Faculdade de Farmácia, Universidade Federal do Rio de Janeiro, Rio de Janeiro, Rio de Janeiro, Brazil; 3 Instituto Federal do Rio de Janeiro (IFRJ), Unidade Nilópolis, Nilópolis, Rio de Janeiro, Brazil; University of Illinois at Chicago, United States of America

## Abstract

**Background:**

Clotrimazole is an azole derivative with promising anti-cancer effects. This drug interferes with the activity of glycolytic enzymes altering their cellular distribution and inhibiting their activities. The aim of the present study was to analyze the effects of clotrimazole on the growth pattern of breast cancer cells correlating with their metabolic profiles.

**Methodology/Principal Findings:**

Three cell lines derived from human breast tissue (MCF10A, MCF-7 and MDA-MB-231) that present increasingly aggressive profiles were used. Clotrimazole induces a dose-dependent decrease in glucose uptake in all three cell lines, with K_i_ values of 114.3±11.7, 77.1±7.8 and 37.8±4.2 µM for MCF10A, MCF-7 and MDA-MB-231, respectively. Furthermore, the drug also decreases intracellular ATP content and inhibits the major glycolytic enzymes, hexokinase, phosphofructokinase-1 and pyruvate kinase, especially in the highly metastatic cell line, MDA-MB-231. In this last cell lineage, clotrimazole attenuates the robust migratory response, an effect that is progressively attenuated in MCF-7 and MCF10A, respectively. Moreover, clotrimazole reduces the viability of breast cancer cells, which is more pronounced on MDA-MB-231.

**Conclusions/Significance:**

Clotrimazole presents deleterious effects on two human breast cancer cell lines metabolism, growth and migration, where the most aggressive cell line is more affected by the drug. Moreover, clotrimazole presents little or no effect on a non-tumor human breast cell line. These results suggest, at least for these three cell lines studied, that the more aggressive the cell is the more effective clotrimazole is.

## Introduction

Among the physiological hallmarks of cancer, altered glucose metabolism is perhaps the most common. The Warburg effect has been observed in approximately 90% of human tumors and the biochemical origins of this phenomenon have been extensively studied [1]–[4]. Aerobic glycolysis may be required for new biomass formation [5]. In fact, proliferation of cancer cells is accompanied by activation of glycolysis [6], which occurs even at normal oxygen concentrations. Moreover, glycolysis may confer tumor cells with the ability to adapt to new microenvironments or cope with stress during tumor progression and metastasis [7].

Observations suggest that blocking glycolysis might diminish tumor progression and enhance the efficacy of chemo- and radiotherapy. However, inhibition of glycolytic enzymes is expected to have secondary effects on cell physiology, due to the additional functions of these proteins. Clotrimazole, an antifungal drug, has been successfully used to diminish the size and development of intracranial gliomas (C6 and 9L), prolonging survival in rodents [8]. Moreover, the drug also affects glycolytic enzymes decreasing hexokinase (HK) binding to the outer mitochondrial membrane [9] and detaching phosphofructokinase-1 (PFK-1) and aldolase from the cytoskeleton [10]–[12]. Indeed, clotrimazole is able to trigger apoptosis, which is directly correlated with its ability to displace HK from mitochondria [9] and PFK-1 and aldolase from the cytoskeleton [12]. The location of these glycolytic enzymes within the intracellular milieu is an important feature of glycolysis regulation [13] and thus, altering the intracellular distribution of these enzymes, clotrimazole is probably affecting the glycolytic flux.

The aim of this study was to analyze the effects of the clotrimazole on viability, growth, mobility and glycolytic profile of three human breast cell lines: MCF10A, MCF-7 and MDA-MB-231. The MCF10A human mammary epithelial cell is a normal strain, while MCF-7 and MDA-MB-231 cells are human breast-derived cell lines with tumorigenic and metastatic profiles, respectively. Here we present evidences that clotrimazole presents more pronounced effects on the tumorigenic and metastatic cells, while presenting minimal effects over the non-tumoral cell strain.

## Results

### Clotrimazole inhibits the migratory phenotype in breast cancer cells

**Figure 1:**
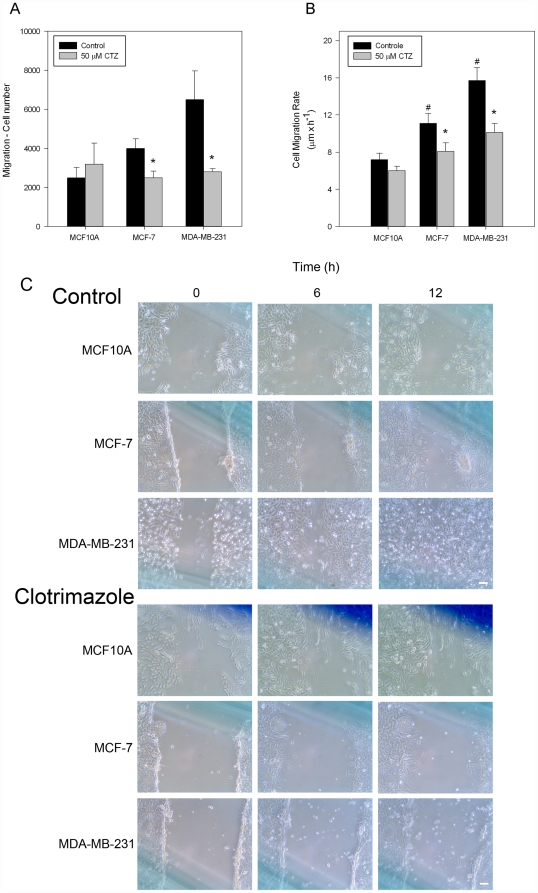
Clotrimazole inhibits the migration of breast cell lines. (A) The migration of MCF10A, MCF-7 and MDA-MB-231 cells was assessed by transwell assays after 24 h incubation in the absence or presence of 50 µM clotrimazole; (B) Cell migration rate was calculated from scratch assays. The images were analyzed and data were expressed in µm×h^−1^. (C) Scratch migration assay for control and 50 µM clotrimazole-treated cells. The filling of scratchs by migrated cells at time 0, 6 and 12 h was imaged. MCF-7 and MDA-MB-231 cells have a more motile appearance and migrate faster than MCF10A. Bars are mean ± SEM of three independent experiments. * P<0.05 compared with control condition in the absence of the drug; ^#^ P<0.05 compared with MCF10A cells.

To assess the effects of clotrimazole on the growth profiles of breast cell lines, cellular migration and proliferation were evaluated. The migration potential of MCF10A, MCF-7 and MDA-MB-231 cells was initially assessed using the Transwell assay (Fig. 1A). Our results confirm that there is a significant difference between the migration potential of these cells, which increases with the aggressiveness of the cell (MCF10A<MCF-7<MDA-MB-231). The presence of 50 µM clotrimazole inhibits migration of MCF-7 and MDA-MB-231 cells, but has no effect on MCF10A cells. Proportionally, the effects of clotrimazole were more pronounced on MDA-MB-231 migration (59±6% inhibition) than on MCF-7 (32±5% inhibition). Moreover, using the scratch assay, we compared cell mobility in the presence or absence of 50 µM clotrimazole (Fig. 1B and 1C). In accordance with the previous results, there is a significant difference between the migration rates of the cell lines assessed through this later technique. The evaluated migration increased approximately 1.5-fold and 2- to 3-fold for MCF-7 and MDA-MB-231 cells, respectively, when compared with control cells (black bars, Fig. 1B). Within 12 hours, the migration rate of the tumorigenic cells was significantly reduced by clotrimazole (grey bars, Fig. 1B). Clotrimazole inhibited mobility of MDA-MB-231 cells by 36±4%, of MCF-7 cells by 16±3%, but was unable to affect the MCF10A migration rate (Fig. 1B and 1C). These results suggest that clotrimazole prevents cell migration, which may contribute to the anti-metastatic potential of the drug.

**Figure 2:**
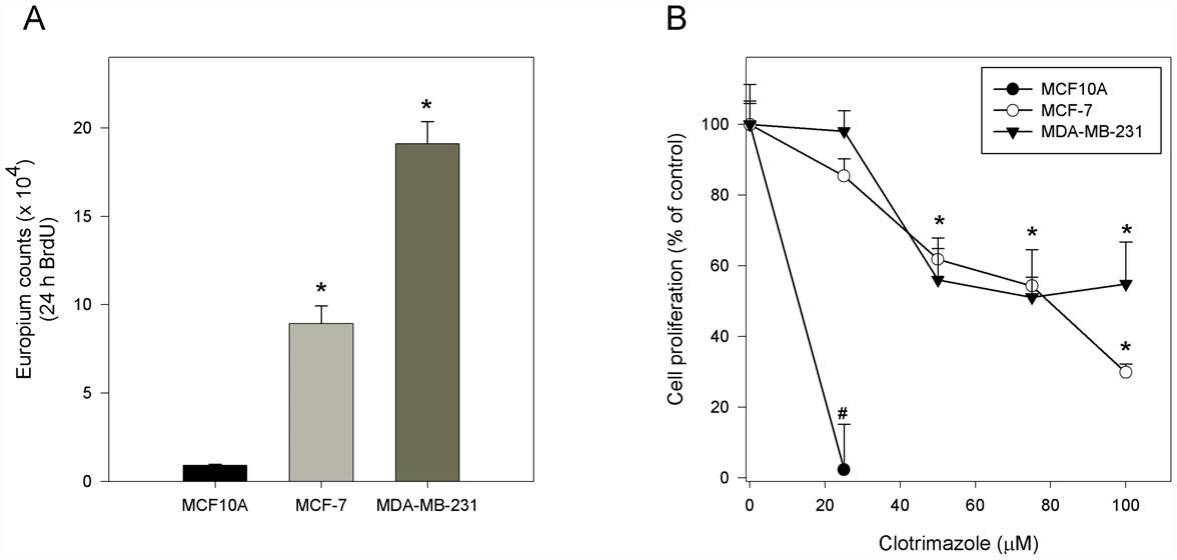
Clotrimazole decreases cell proliferation of MCF10A, MCF-7 and MDA-MB-231 cells. (A) Basal cell proliferation of human breast cell lines was analyzed using the BrdU incorporation assay. The graph shows the europium-based TRF cell proliferation assay. * P<0.05, in comparison with MCF10A cell line. (B) Effects of clotrimazole on cell proliferation after 24 h treatment using BrdU incorporation assay. Values are mean ± standard error (SE) of four different experiments. ^#^ P<0.05, in comparison with the control; * P<0.05, comparing 50, 75 and 100 µM clotrimazole with the control in the absence of the drug for both cell lines (MCF-7 and MDA-MB-231).

To clarify that these results were not due to the effect of clotrimazole on the cell proliferation, we determined the incorporation of BrdU into DNA of synchronized proliferating breast cell lines in the absence or presence of different concentration of clotrimazole. Fig. 2A shows cell proliferation in the absence of clotrimazole after 24 h of BrdU incubation. MCF-7 and MDA-MB-231 cells display a 10- and 20-fold higher proliferation rate, respectively, when compared with the MCF10A cells. Treatment with clotrimazole (0–100 µM) inhibited the cell proliferation in all cell lines (Fig. 2B). Twenty-four hours after cell cycle reentry, which is initiated by adding fresh culture medium containing fetal serum, the percentage of proliferating cells was significantly decreased by clotrimazole. Surprisingly, 25 µM clotrimazole completely inhibited MCF10A proliferation (98±13% inhibition). However, this same concentration had no effect on tumoral cell lines. MCF-7 cells displayed a linear response to increasing clotrimazole concentrations, reaching 70% inhibition at 100 µM. Furthermore, clotrimazole also inhibited proliferation of MDA-MB-231 cells, but the maximum inhibition (50%) occurred at 50 µM and persisted even at the highest concentration tested (100 µM).

### Clotrimazole diminishes glucose uptake and intracellular ATP

**Figure 3:**
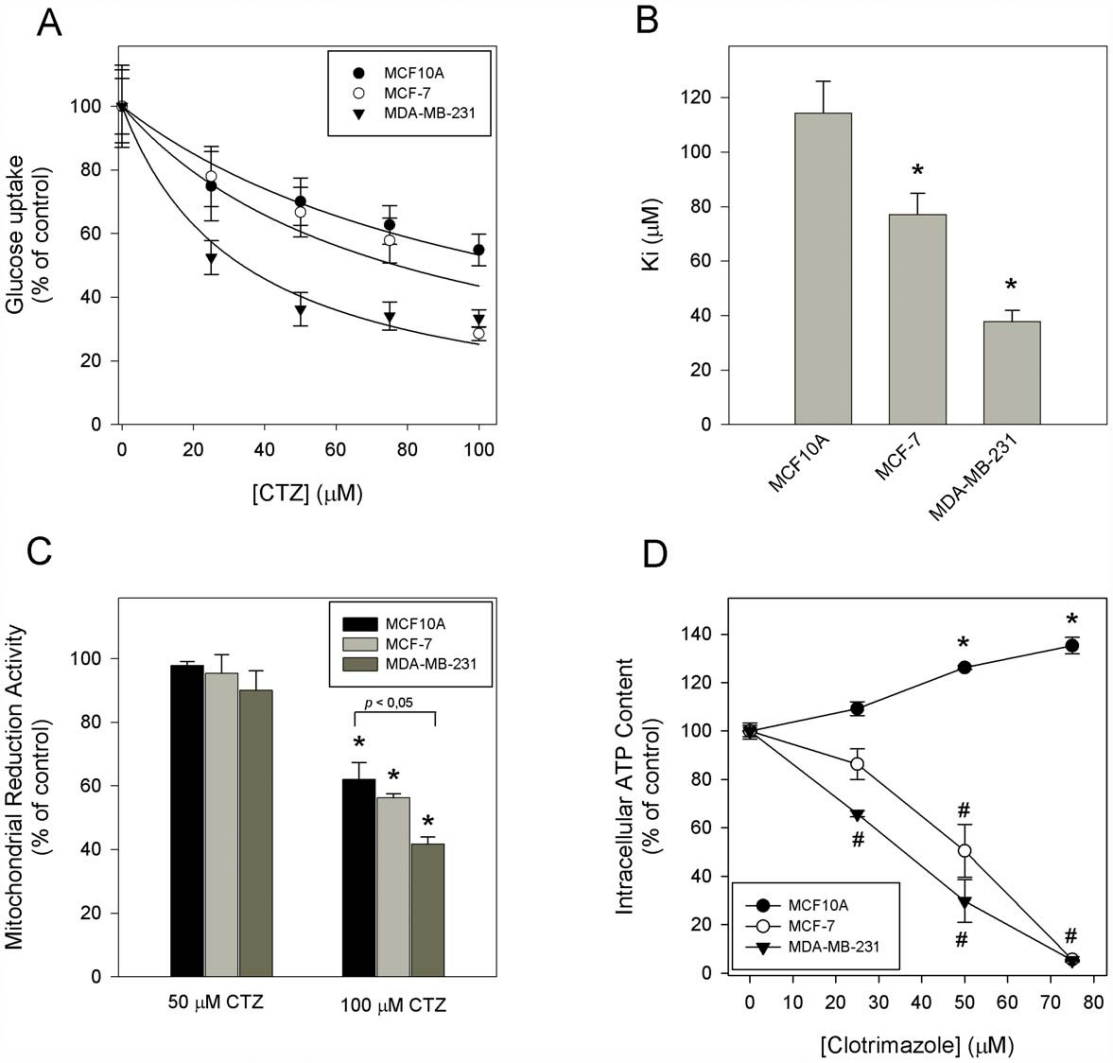
Effects of clotrimazole on glucose uptake, mitochondrial reduction activity and cellular ATP content in breast cell lines. (A) Comparison of glucose uptake in MCF10A, MCF-7 and MDA-MB-231 cells. Glucose uptake was determined after 15, 30 and 45 min incubation through cells incubation with 5 mM 6-NBDG, a fluorescent glucose analogue. The results obtained are plotted as percentage of control in a function of clotrimazole concentration. (B) Glucose uptake experimental data was fitted in an equation as described in Methods and the K_i_ values were plotted. Bars are mean ± SEM of three independent experiments. * P<0.05, comparing with MCF10A cells. (C) Percent of mitochondrial reduction activity, evaluated by MTT assay. All values were normalized to that of control condition in the absence of the drug. (D) Intracellular ATP content measured by relative firefly luciferase activity (PerkinElmer ATPLite Kit). Error bars represent standard errors from five independent experiments. * P<0.05 compared to control for MCF10A cells. ^#^ P<0.05, compared to control for MCF-7 and MDA-MB-231 cell lines.

Proliferation in cancer cells is accompanied by activation of glycolysis [6], which occurs even in the presence of a normal oxygen concentration. This preferential use of glycolysis by cancer cells under aerobic conditions is known as the Warburg effect [14] and is characterized by high glycolytic rates in cancer cells with high rates of lactate production per glucose consumed. Our previous results have shown that breast cancer cells present a distinct glycolytic rate; the metastatic cell line (MDA-MB-231) showed lower consumption of glucose and higher production of lactate when compared to a non-tumorigenic cell line (MCF10A). Moreover, MCF-7 cells, which are tumorigenic and non-metastatic, presented an intermediate glycolytic rate [15]. In order to evaluate the metabolic profiles of these breast cell lines after treatment with clotrimazole, we incubated the cells in 96 well plates with different concentrations of clotrimazole (0–100 µM) for 24 h. Clotrimazole reduces glucose uptake by the cells in a dose-dependent fashion (Fig. 3A). The inhibition constant (K_i_) for glucose uptake inhibition by clotrimazole was calculated and found to be 114.3±11.7, 77.1±7.8 and 37.8±4.2 µM for MCF10A, MCF-7 and MDA-MB-231 cells, respectively (Fig. 3B). These results clearly demonstrate that clotrimazole has more pronounced effects on cells with the highest glycolytic rates and Warburg effect.

We also analyzed the mitochondrial reduction activity and the cellular ATP levels after the treatment with clotrimazole. Fig. 3C shows a dose-dependent reduction of the mitochondrial activity in these cells, with the maximum inhibition being approximately 60% for MDA-MB-231 cells treated with 100 µM clotrimazole. At that concentration, both MCF10A and MCF-7 cell lines demonstrate 40% lower mitochondrial activity than corresponding cells treated with 50 µM clotrimazole. Once more, the drug was more effective in the metastatic cell line than the non-tumoral cells in the same condition (Fig. 3C). Furthermore, we evaluated the effects of clotrimazole on the cellular ATP levels. Clotrimazole reduces the cellular ATP of tumorigenic cells in a dose-dependent manner (Fig. 3D). Interestingly, MCF10A cells are completely resistant to the presence of the drug. In fact, we observed 26% and 35% higher ATP content after the treatment with 50 and 75 µM clotrimazole, respectively. However, MCF-7 and MDA-MB-231 cells display a progressive dose-dependent inhibition curve reaching 95% reduction of the ATP content with 75 µM clotrimazole.

### Glycolytic enzymes from tumorigenic cells are selectively inhibited by clotrimazole

The results above show that clotrimazole alters the rates of glucose uptake, mitochondrial activity and generation of ATP in the cell lines, with more pronounced effects on the cancerous cell lines than non-tumorigenic cells. Additionally, these effects probably are not due to a specific action of clotrimazole on mitochondrial activity because all the cell lines, regardless of their phenotype, are not responsive to clotrimazole up to 100 µM (Fig. 3C). Therefore, our next objective was to investigate whether the antineoplasic effects of clotrimazole occurs by its direct action on glycolysis. Therefore, we evaluated the effects of the drug on the three enzymes that regulate the glycolytic flux: hexokinase (HK), phosphofructokinase-1 (PFK-1) and pyruvate kinase (PK).

**Figure 4:**
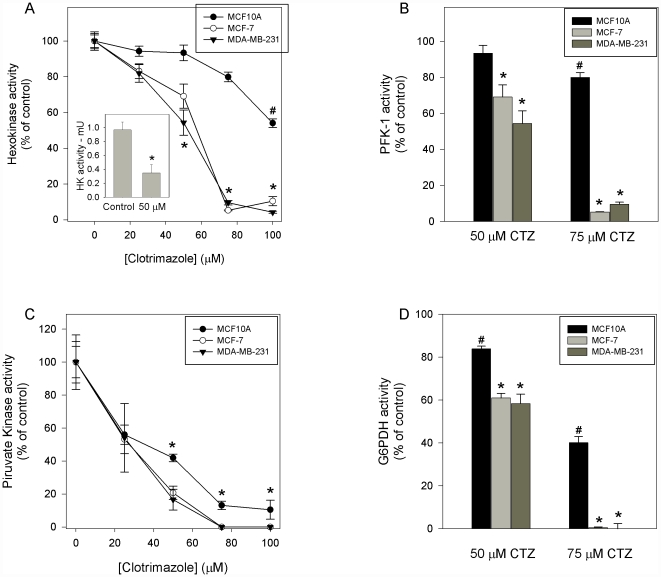
Glycolytic enzymes activity and G6PDH activity are inhibited by clotrimazole. Cell lines were grown to confluence in the indicated media as described in Methods. Cell lysates were used to evaluate HK, PFK-1, PK and G6PDH activities (panel A, B, C and D respectively) as described in Methods. Plotted values are mean ± standard errors of five independent experiments. (A) ^#^ P<0.05 compared to control in the absence of clotrimazole; * P<0.05, compared to control in the absence of clotrimazole. (B) ^#^ P<0.05 compared to control in the absence of clotrimazole; * P<0.05, compared to control and to MCF10A in the presence of clotrimazole. (C) The differences among the results obtained with the distinct clotrimazole concentrations tested are statistically significant. * indicate differences between MCF10A and tumoral breast cell lines. (D) ^#^ P<0.05 compared to control in the absence of clotrimazole; * P<0.05, compared to control and to MCF10A in the presence of clotrimazole.

We evaluated HK activity in all breast cells using a 24 h incubation time with different concentrations of clotrimazole. Fig. 4A shows HK dose-dependent inhibition curves for MCF10A, MCF-7 and MDA-MB-231 cells, where clotrimazole has a more pronounced effect in tumoral cell lines compared to the non-tumoral cells. HK activity in MCF10A cells was inhibited by only 46% by clotrimazole, whereas, at the same concentration (100 µM clotrimazole), HK activity in MCF-7 and MDA-MB-231 cells was inhibited by 90% and 96%, respectively. To align our results with the data in the literature, we used the 3-bromopyruvate (3-BrPA), a well-known inhibitor of energy metabolism, which has been proposed as a specific anticancer agent due to its high tumor selectivity [16], [17]. Surprisingly, 3-BrPA has different effects on these cell lines, showing a more pronounced inhibition of HK in the MCF10A cells (Table 1). This is an interesting result because, until now, both drugs (3-BrPA and clotrimazole) were thought to have the same mechanism of action in cancer cells: through the detachment of the mitochondria-associated HKII. Although this is an important mechanism to cell survival, our results strongly suggest an additional mechanism of cell death induced by the direct inhibition of HK by clotrimazole (Fig. 4A, inset).

**Table 1:** 3-BrPA effects on HK activity from breast cell lines.

Cell line	3 h treatment with 100 µM 3-BrPA(% of control)
**MCF10A**	74.2±4.9
**MCF-7**	86.6±0.6
**MDA-MB-231**	105.9±1.9

Meira and co-workers showed that clotrimazole inhibits phosphofructokinase-1 in MCF-7 cells [12]. To investigate this inhibition further, we analyzed the effects of clotrimazole on breast cell lines. Similar to what was observed for HK, clotrimazole inhibits PFK-1 activity in a dose-dependent manner (Fig. 4B). The effects of clotrimazole are dependent on the tumorigenic phenotype of the cell, as MCF10A cells are more resistant to the inhibition than MCF-7 and MDA-MB-231 cells. Once again, we observed 90–95% inhibition in tumorigenic cells and a less dramatic effect of clotrimazole on MCF10A cells.

We also evaluated the effects of clotrimazole on the last enzyme of glycolysis, pyruvate kinase (PK). Once more, clotrimazole progressively inhibited enzyme activity, with a substantial effect on MCF-7 and MDA-MB-231 cells (Fig. 4C). However, there was a more significant inhibition of PK in MCF10A cells when compared with the results for HK and PFK in this cell line. This result may be due to the effects of clotrimazole on previous steps of glycolysis or on glucose uptake in these cells.

After entering the cell, glucose is converted by HK to glucose 6-phosphate, which can be shunted to the pentose phosphate pathway (PPP). The increase in the PPP flux is directly associated with inhibition of the apoptosis. This is due to an increased generation of NADPH and, therefore, reduced glutathione, which also promotes the removal of reactive oxygen species (ROS) in cells [18]. To investigate the diversion of glucose 6-phosphate from glycolysis to PPP, we verified the activity of glucose 6-phosphate dehydrogenase (G6PDH), which is the enzyme responsible for the first irreversible reaction of the PPP. Similar to the results observed for the glycolytic enzymes, clotrimazole inhibits the PPP (Fig. 4D). The inhibitory effect of clotrimazole is more pronounced in MCF-7 and MDA-MB-231 cells than in MCF10A cells. The complete inhibition of PPP was obtained with 75 µM clotrimazole for tumorigenic cell lines and 100 µM clotrimazole for the MCF10A cells.

### Metabolic effects of clotrimazole induce the loss of viability

**Figure 5:**
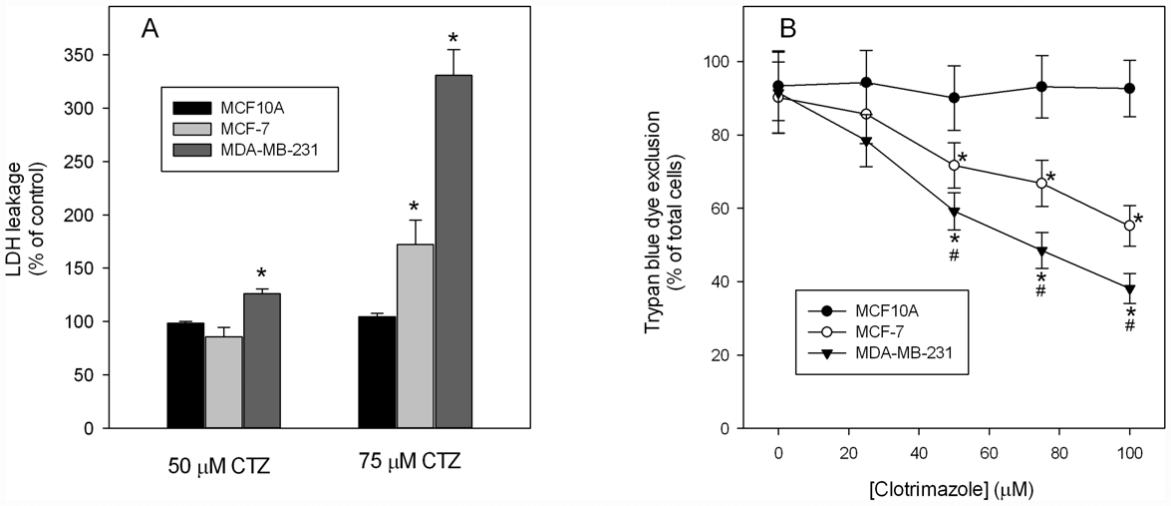
Cellular viability decreases in breast cancer cells treated with clotrimazole. Data are presented as mean ± SE of at least five experiments. Panel A: lactate dehydrogenase (LDH) leaked to culture medium by clotrimazole-induced cellular lyse was evaluated as described in Methods. * P<0.05 compared to MCF10A cells in the same clotrimazole concentration. Panel B: the percentages of cells that exclude trypan blue dye were evaluated counting the total cells and those that were intracellularly stained with the dye. Cells were counted using a TC10 Automated Cell Counter (Bio-Rad Laboratories, CA, USA). * P<0.05 compared to MCF10A in the same clotrimazole concentration. # P<0.05 compared to MCF-7 in the same clotrimazole concentration.

Finally, we investigated the consequences of the metabolism inhibition induced by clotrimazole on cell viability. Published data have shown that clotrimazole reduces the viability of MCF-7 cells, CT-26 colon adenocarcinoma cells and Lewis lung carcinoma [11], [19], as well as B16 melanoma cells [10], [20]. However, as most of our results demonstrate a cellular phenotype-dependent effect of clotrimazole, our objective was to evaluate whether cell viability also responds in a similar way. The cell viability was evaluated by the activity of lactate dehydrogenase (LDH) leaked to the medium and permeability of the cells to trypan blue dye. As shown in Fig. 5A, clotrimazole is does not affect the leakage of LDH of MCF10A cells in any of the concentrations tested, even at 100 µM clotrimazole (data not shown), as compared to untreated controls. This result strongly suggests that the drug does not affect MCF-10A cells integrity. This picture changes when we analyze the effects of clotrimazole on LDH leakage from MCF-7 cells. Although 50 µM clotrimazole does not alter LDH leakage from these cells, 75 µM clotrimazole promotes a significant increase of the leaked enzyme suggesting the loss of cell integrity and viability. On the other hand, MDA-MB-231 cells viability is more susceptible to clotrimazole effects, since this cell line presents increased LDH leakage upon the treatment with 50 µM clotrimazole for 24 hours. Moreover, treatment of MDA-MB-231 cell with 75 µM clotrimazole increases 3.5 times the leaked LDH suggesting that the viability of these cells is severely compromised upon this treatment. These data are confirmed analyzing the exclusion of trypan blue dye by the different cell lines treated with clotrimazole (Fig. 5B). These results corroborate that MCF10A cells integrity is not affected up to 100 µM clotrimazole. Moreover, it is clear now that upon the treatment with clotrimazole the viability of MCF-7 and MDA-MB-231 cells decrease in a dose-responsive mode. Furthermore, MDA-MB-231 cells are more sensitive to 50 µM clotrimazole and higher concentrations, as compared to MCF-7 cells. Altogether, these data reinforce the preferential action of clotrimazole over the tumorigenic cell lines among those tested here, with an even more preferential effect over the most aggressive cell line.

## Discussion

The use of metabolic pathways as useful molecular targets in tumors prompted us to investigate the effects of drugs that affect cell metabolism, especially glycolysis. In this paper, we report the metabolic and anti-metastatic effects of the imidazole derivative clotrimazole in breast cell lines. Our data reveal that clotrimazole has more pronounced effects on tumorigenic cell lines that present the Warburg effect [15]. There are, at least, three major possible explanations to the preferential effects of clotrimazole on tumorigenic cell lines: (1) cancerous cells accumulate clotrimazole more readily and in an aggressiveness-dependent pattern, (2) glycolytic enzymes in cancerous cells are more sensitive to the effects of clotrimazole what could be due to an isoform expression specificity, and (3) cancerous cells rely on glycolysis more heavily and are therefore more sensitive, which is strongly supported by the Warburg effect observed in tumoral and not in non-tumoral cells. The first possibility is reasonably plausible, but we have not evaluated it and therefore we cannot discuss it. The other two possibilities seem to be supported by our results, although we cannot unambiguously discriminate between them.

In non-tumoral cells, at least 90% of ATP production is provided by mitochondrial oxidative phosphorylation, while in tumor cells, approximately 50% is dependent on cytoplasmic, aerobic glycolysis [21], [22]. Here, we demonstrate that clotrimazole inhibits glucose uptake (Fig. 3A) and mitochondrial activity (Fig. 3C) more efficiently in the tumorigenic cell lines. These effects result in alterations in ATP content that are also more pronounced in the tumorigenic cell lines. However, it seems that this later effect is more related to the inhibition of glycolysis, since mitochondrial activity is not reduced by 50 µM clotrimazole, while glucose consumption and ATP content are affected by even lower concentrations of the drug. These data highlight the importance of aerobic glycolysis for energy production in tumorigenic cells. The increased content of ATP measured in MCF-10A cell upon clotrimazole treatment might be the result of clotrimazole effects on this cell metabolism and proliferation. Comparing clotrimazole effects on cells proliferation and metabolism one can observe that the drug blocks the proliferation of MCF-10A cells (Fig. 2B), decreases its glucose consumption (Fig. 3A) and do not affect its mitochondrial activity (Fig. 3C). Similar results are observed for the tumorigenic cell lines but with lesser extent on proliferation and greater extent on metabolism. Since proliferation if the highest ATP-consuming cellular process, the resultant ATP content of MCF-10A cells increases upon clotrimazole treatment.

It is important to mention that the lack of clotrimazole effects on MCF10A cell line cannot be attributed to fact that this cell line is grown in a different medium (DMEM/F12 with 5% FHS, instead of DMEM with 10% FBS used for MCF-7 and MDA-MB-231). We have performed control experiments assaying the effects of clotrimazole on cell viability and enzymes activity of MCF-10A cells grown in DMEM/F12 but incubated with DMEM for 24 hours during the clotrimazole treatment and the results were not different from those presented here. We decided to perform all the experiments using DMEM/F12 for the MCF10A cell to assure that all the cells would be at optimal growing conditions (MCF-10A does not grow well in DMEM with 10% FBS).

Many human tumors display a high rate of aerobic glycolysis, de novo fatty acid synthesis, and nucleotide biosynthesis [23], [24]. Previous findings suggest that the increased glucose metabolism promotes lipogenesis and nucleotide biosynthesis, and enhances tumor cell growth and proliferation by providing essential synthetic and bioenergetic requirements [25]–[29]. Although the metabolic alterations might not be initiating events in oncogenesis, recent success in blocking carcinogenesis by targeting tumor metabolism suggests that aerobic glycolysis plays an important role in sustaining tumor growth [27]–[33].

After 24 hours of incubation, clotrimazole promote a phenotype-dependent inhibition of the key glycolytic enzymes: HK, PFK-1 and PK. The results always present the same pattern: MCF10A cells are less responsive to clotrimazole, whereas MCF-7 and MDA-MB-231 are sensitive to the increasing concentration of the drug (Fig. 4). More importantly, the metastatic cell line presents the highest clotrimazole-induced inhibition of glycolysis. These observations are accompanied by the dose-dependent inhibition of G6PDH, the enzyme responsible for the first irreversible reaction of the PPP (Fig. 4D). We have not observed differences between the effects of clotrimazole on enzymes activity of MCF-7 and MDA-MB-231 cell lines. The drug appears to be more effective inhibiting tumoral than non-tumoral enzymes, without distinction on the aggressiveness of the tumoral cell line. However, this can be an underestimated result, since here we measured the whole enzyme activity. These measurements were achieved under optimal substrate concentration and other reaction conditions, which can mask some fine regulatory properties of these enzymes, such as their association with cellular macromolecular structures, described as important to cancer increased glycolytic rates [34]. However, in order to assess these possibilities, a profound study of the effects of clotrimazole on glycolytic enzyme kinetics and cellular distribution should be performed, which was not the objective of the current work.

Although cancer cells are capable of carrying out oxidative phosphorylation, they undergo the glycolytic shift because aerobic glycolysis is advantageous for cell proliferation and tumorigenicity [35]. The metabolism of cancer cells, and indeed all proliferating cells, is adapted to facilitate the uptake and incorporation of nutrients into the biomass needed to produce a new cell. Supporting this idea are recent studies showing that (i) several signaling pathways implicated in cell proliferation also regulate metabolic pathways that incorporate nutrients into biomass; and that (ii) certain cancer-associated mutations enable cancer cells to acquire and metabolize nutrients in a manner conducive to proliferation rather than efficient ATP production [5]. Moreover, in metastases, the glycolytic phenotype persists due to increased production of glucose-derived acid, which potentiates invasiveness of cells into the extracellular matrix of the host tissue [36].

Our results here demonstrated that clotrimazole was able to decrease cancer cells proliferation and viability, with minimal effects on non-tumoral cells. Clotrimazole has been implicated in other mechanisms affecting tumor cells migration and invasion, such as the inhibition of the Ca^2+^-activated potassium channels [37], [38] and inhibition of the enzymes of the cytochrome P450 family [39]. This latter effect of clotrimazole is critically important and represents a reason for why clotrimazole is practically not used for systemic treatments due to its impact on liver CYP450 metabolism. However, clotrimazole have been used to design more selective and less toxic compounds, such as TRAM-34 [40], which, in addition to clotrimazole, was recently demonstrated to inhibit glioblastoma proliferation [41]. An interesting observation is that the effects of clotrimazole and its analogue TRAM-34, originally designed as a specific Ca^2+^-activated potassium channels blocker, on glioblastoma proliferation are not due to any effects on this ion channel. It is not difficult to imagine that the effects of these drugs might be related to inhibition of glioblastoma metabolism, such as described here. Interestingly, intracranial administration of clotrimazole has been used to prolong the survival of rats with glioma, but no mechanisms have been reported for this effect [8]. Clotrimazole has been shown to induce the nuclear condensation and f-actin depolymerization in MCF-7 cells [12] suggesting that clotrimazole-treated cells undergo apoptosis. In fact, decrease in glycolytic flux has been associated elsewhere to some apoptotic events [42], [43]. In addition, studies on cell metabolism during apoptosis in murine interleukin-3-dependent cell line revealed that the activation of glycolytic flux is related to cell survival [42]. However, we didn't yet explore the death mechanisms involved in the decrease of cellular viability in response to clotrimazole on tumor cells.

Our current results add to the growing evidence that aerobic glycolysis contributes to cancer cell proliferation and tumorigenicity. Although the systemic therapeutic use of clotrimazole is strongly limited due to its toxic effects, the present findings point to the development of novel treatment approaches that selectively targets glycolysis in tumor cells. These novel approaches may identify treatments that would be more selective to aggressive tumors with minimal effects over non-tumoral cells.

## Materials and Methods

### Cell lines and Materials

The human breast cancer cell line MCF-7 was obtained from the Cell Bank of Hospital Universitário Clementino Fraga Filho, UFRJ, Brazil. MDA-MB-231 and the non-tumorigenic epithelial MCF10A cell line were kindly supplied by Prof. Dr. Mitzi Brentani from University of São Paulo, USP, Brazil. The tumor cell lines were maintained in DMEM (Dulbecco's modified Eagle's medium; Invitrogen) supplemented with 10% (v/v) FBS (fetal bovine serum; Invitrogen) and L-glutamine. The MCF10A cell line was maintained in DMEM/F12 medium (Invitrogen) supplemented with 5% FHS (fetal horse serum; Gibco) plus 0.02 µg/ml EGF, 5 µg/ml insulin, 1.25 µg/ml hydrocortisone and 0.1 µg/ml cholera toxin (Sigma Chemical, St. Louis, MO, USA). Cells were grown at 37°C in 5% CO_2_ atmosphere.

Clotrimazole, 3-bromopyruvate, NAD^+^, NADH, ATP, hexokinase, glucose 6-phosphate dehydrogenase, fructose 6-phosphate, aldolase, lactate dehydrogenase, phosphoenolpyruvate, NADP^+^ and glucose 6-phosphate were obtained from Sigma. Other reagents were of the highest purity available.

### Cell viability assay, glucose uptake, mitochondrial activity and ATP quantitative evaluation

To measure metabolic rates, cells were seeded in 96-well plates in the appropriate medium and grown to confluence. Then, the medium was removed, fresh medium was added, and the cells were returned to the incubator in the presence of different concentrations of clotrimazole (0–100 µM). After 24 h, the medium was removed and the amount of leaked lactate dehydrogenase (LDH) was evaluated, while mitochondrial activity and trypan blue dye exclusion was determined in the remaining cells using an MTT assay [44], [45] and counting the cells that exclude trypan blue [46], respectively.

Glucose uptake was determined by incubating cells with fresh medium containing 5 mM 6-(N-(7-nitrobenz-2-oxa-1,3-diazol-4-yl)amino)-6-deoxyglucose (6-NBDG, Molecular Probes, Invitrogen, Life Technologies, Carisbad, CA, USA), a fluorescent glucose analogue. After 15, 30 and 45 min incubation, the medium was removed, the cells were washed with 6-NBDG-free medium, and 6-NBDG taken up by the cells was evaluated by fluorescence emission, according to the manufacturer's instructions. The glucose uptake rate was calculated by the linear regression of the increase in fluorescence incorporated by the cells, and these results are plotted as a percentage of control in a function of clotrimazole concentration. The parameters of the following equation were fitted to the experimental data in order to evaluate the effectiveness of clotrimazole effects by means of the concentration promoting half of the maximal glucose uptake inhibition. The equation used was:
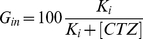
(1)where *G_in_* is the percentage of glucose taken up by the cells at a given concentration of clotrimazole ([*CTZ*]) and *Ki* is the inhibitory constant, equivalent to the concentration of clotrimazole promoting 50% of the maximal inhibition.

LDH activity was determined spectrophotometrically by monitoring the conversion of NAD^+^ to NADH in a lactate-utilizing reaction [47]. Cellular ATP was assessed using a system based on firefly (Photinus pyralis) luciferase (PerkinElmer ATPLite) using a VICTOR3 multilabel reader (PerkinElmer).

### Spectrophotometric assay for enzymes activity

Cells were seeded in 24-well plates and grown to confluence. Then, medium was removed and fresh medium was added, and cells were returned to the incubator in the presence of different concentrations of clotrimazole (0–100 µM) for 24 h. After this incubation, cells were removed from the plates by trypsinization and counted using a hemocytometer. Protein concentrations of cell lysates were measured [48], and the glycolytic enzyme activities were evaluated.

Phosphofructokinase-1 (PFK-1) activity was assayed using an enzyme-coupled method, where the oxidation of NADH was monitored spectrophotometrically [49]. The basic medium contain 50 mM Tris-HCl (pH 7.4), 5 mM MgCl_2_, 1 mM fructose 6-phosphate, 1 mM ATP, 0.2 mM NADH, 2 U/ml aldolase, 4 U/ml triosephosphate isomerase and 2 U/ml α-glycerophosphate dehydrogenase.

Hexokinase (HK) activity was assessed in a basic medium containing 50 mM Tris-HCl (pH 7.4), 5 mM MgCl_2_, 1 mM glucose, 1 mM ATP, 0.2 mM NAD^+^ and 1 U/ml glucose 6-phosphate dehydrogenase as described previously [50].

Pyruvate kinase (PK) activity was measured in a medium containing 50 mM Tris-HCl (pH 7.4), 5 mM MgCl_2_, 120 mM KCl, 1 mM phosphoenolpyruvate, 1 mM ADP, 0.2 mM NADH and 20 U/ml lactate dehydrogenase as described previously [50].

Glucose-6-phosphate dehydrogenase (G6PDH) activity was assayed as previously described [51]. The basic medium contains 50 mM Tris-HCl (pH 7.4), 5 mM MgCl_2_, 0.5 mM glucose 6-phosphate and 0.2 mM NADP^+^.

For all enzymatic analyses, NADH oxidation or NAD(P)^+^ reduction was determined by measuring the absorbance at 340 nm in a microplate reader (VICTOR 3, PerkinElmer). Reactions were initiated by the addition of an aliquot of cellular homogenate and followed online during the first-order kinetics period. Enzymes rates was determined calculating the first derivative of these curves. Blanks containing none of the coupled enzymes (or without glucose 6-phosphate in the case of G∧PDH) were performed to control for non-specific oxidation/reduction. Each curve was performed in quadruplicate.

### Cell migration assays

Cell migration was determined by using the 24-well Millicell Hanging Cell Culture Insert (Millipore) chamber with 8 µm pore polyethylene terephthalate (PET) membranes. The chambers were rehydrated in serum-free medium. Complete medium with 10% FBS or FHS was used as a chemoattractant. Suspension of 5×10^4^ cells/ml in serum-free medium was added to the inserts and incubated for 24 h in the presence or absence of 50 µM clotrimazole. Cells remaining on the upper membrane surface of the inserts were removed with a cotton swab whereas the cells on the lower surface were trypsinized and counted.

Cell migration was also assessed in scratch assays [52]. Briefly, confluent MCF10A, MCF-7 and MDA-MB-231 cells plated on tissue culture dishes were manually scratched with a 200 µl pipette tip, washed with PBS and incubated at 37°C in complete media in the presence or absence of 50 µM clotrimazole. At the indicated time points, phase contrast images at specific scratch sites were captured as previously described [53]. The images were analyzed using the software BELView (BEL Engineering, It) and data were expressed in µm×h^−1^.

### Cell proliferation assay

Cells were seeded in 96-well culture plates and grown until confluence was reached. After 72 h serum was withdrawn to synchronize the cell cycle, cells were evaluated for proliferation in the presence of different concentrations of clotrimazole (0–100 µM) and 10 µM 5-bromo-2′-deoxyuridine (BrdU). BrdU incorporation was analyzed after 24 h of incubation using a europium-based time-resolved fluorescence (TRF) cell proliferation assay according to the manufacturer's instruction (PerkinElmer). Cells incubated without BrdU served as the negative control. The mean fluorescence for each group was determined after subtracting the mean value of the negative control from each cell.

### Statistical analyses

Statistical analysis and non-linear regression was performed using SigmaPlot 10.0 software integrated with SigmaStat 3.1 package (Systat, CA, USA). Student's t-test was used unless otherwise indicated. *p* values≤0.05 were considered statistically significant.
